# A Genome-Wide Expression Profile of Salt-Responsive Genes in the Apple Rootstock *Malus zumi*

**DOI:** 10.3390/ijms141021053

**Published:** 2013-10-18

**Authors:** Qingtian Li, Jia Liu, Dunxian Tan, Andrew C. Allan, Yuzhuang Jiang, Xuefeng Xu, Zhenhai Han, Jin Kong

**Affiliations:** 1 College of Agronomy and Biotechnology, China Agricultural University, Beijing 100193, China; E-Mails: pinkcarlton@126.com (Q.L.); jyz0112@gmail.com (Y.J.); wl1808@sina.com (X.X.); han20131133@hotmail.com (Z.H.); 2 Horticulture Research Institute, Sichuan Academy of Agricultural Sciences, Chengdu 610066, Sichuan, China; E-Mail: liumengjia.822@163.com; 3 Department of Cellular & Structural Biology, the UT Health Science Center, San Antonio, TX 78229-3900, USA; E-Mail: Tan@uthscsa.edu; 4 Plant & Food Research, Private Bag 92169, Auckland, New Zealand; E-Mail: Allan1212121@gmail.com

**Keywords:** salt stress, *Malus zumi*, microarray, salt-responsive gene, interaction network

## Abstract

In some areas of cultivation, a lack of salt tolerance severely affects plant productivity. Apple, *Malus* x *domestica* Borkh., is sensitive to salt, and, as a perennial woody plant the mechanism of salt stress adaption will be different from that of annual herbal model plants, such as *Arabidopsis. Malus zumi* is a salt tolerant apple rootstock, which survives high salinity (up to 0.6% NaCl). To examine the mechanism underlying this tolerance, a genome-wide expression analysis was performed, using a cDNA library constructed from salt-treated seedlings of *Malus zumi*. A total of 15,000 cDNA clones were selected for microarray analysis. In total a group of 576 cDNAs, of which expression changed more than four-fold, were sequenced and 18 genes were selected to verify their expression pattern under salt stress by semi-quantitative RT-PCR. Our genome-wide expression analysis resulted in the isolation of 50 novel *Malus* genes and the elucidation of a new apple-specific mechanism of salt tolerance, including the stabilization of photosynthesis under stress, involvement of phenolic compounds, and sorbitol in ROS scavenging and osmoprotection. The promoter regions of 111 genes were analyzed by PlantCARE, suggesting an intensive cross-talking of abiotic stress in *Malus zumi.* An interaction network of salt responsive genes was constructed and molecular regulatory pathways of apple were deduced. Our research will contribute to gene function analysis and further the understanding of salt-tolerance mechanisms in fruit trees.

## 1. Introduction

Salt stress severely affects crop production worldwide. Efforts have been made to uncover the mechanisms of salt tolerance in model plants using molecular and genomic approaches. Many salt tolerant genes have been identified [1,2], which release plants from salt stress through stress perception, signal transduction and transcriptional regulation for cellular responses including ROS scavenging, osmolyte accumulation, and transportation of water and ions through the plasma membrane and tonoplast [3,4]. The over-expression of a number of salt-induced genes confers stress tolerance to the transgenic plants [5,6].

Three primary signaling pathways in salt-stress response (CDPK, CIPK/SOS, and MAPK pathways) have been reported in plants [4]. The Ca^2+^-dependent signaling (CDPK pathway) is extensively studied, which induces the expression of DRE/CRT transcription factors and other types of LEA-like genes. The CIPK/SOS pathway appears to be relatively specific for the ionic transportation, in which high Na^+^ stress initiates a calcium signal that stimulates the SOS3-SOS2 protein kinase complex, which then activates the Na^+^/H^+^ exchange transporter SOS1 and regulates the expression of several other salt-responsive genes. In addition, SOS3-SOS2 may activate or suppress the activities of other transporters involved in Na^+^ homeostasis. The MAPK pathway regulates production of compatible osmolytes and antioxidants, and may also participate in cell cycle regulation under salt stress.

Although the molecular mechanism of salt tolerance has been intensively studied in model plants, in perennial woody species such as apple, the mechanism remains unclear [7,8]. Apple (*Malus* × *domestica* Borkh.) is one of the most valuable horticultural fruit crops in the world. While it produces the highest yield in China, it is subject to severe salt stress in many areas [9,10]. A better understanding of the genetic bases of salt tolerance would contribute to the molecular breeding of salt resistant apples. Rootstocks play a key role in salt tolerance. The increasing secondary salinity threats apple production. Because in the commercial orchards, all the apple cultivars are grated onto rootstocks, which are subjected to the high salinity in the soil. *Malus zumi* is a local rootstock, distributed in the Northeast of China, which can survive high salinity (approximately 0.6% NaCl).

The draft apple genome has been recently published, with the majority of the genes not functionally annotated [11]. Furthermore, apple transcriptomics have been performed using microarray analysis [12], however no analysis or isolation has been made of ESTs from samples under salt stress. To gain a genome-wide view of salt response, a cDNA library was constructed in *Malus zumi* under salt stress by SMART ^™^. A total of 15,000 cDNA clones were selected randomly for microarray analysis, among which, 576 cDNA clones changing their expression significantly (more than four-fold) under stress were sequenced. Our research resulted in novel gene isolation and new molecular insights of apple-specific salt-tolerant mechanisms in term of ROS scavenging, osmoprotection, and photosynthesis in *Malus zumi*. The *cis*-elements in the promoter region of salt-responsive genes were analyzed. A network of salt responsive genes was constructed and molecular regulatory pathways of apple were deduced. This helped to elucidate salt-tolerant mechanisms from a novel perspective. Our research will contribute to identification of genes in response to salt-tolerance and as well as providing new insight into understanding salt-tolerance mechanisms in apple trees.

## 2. Results

### 2.1. Salt-Responsive Genes Identified by Microarray Experiment and Semi-Quantitative RT-PCR Validation

**Figure 1:**
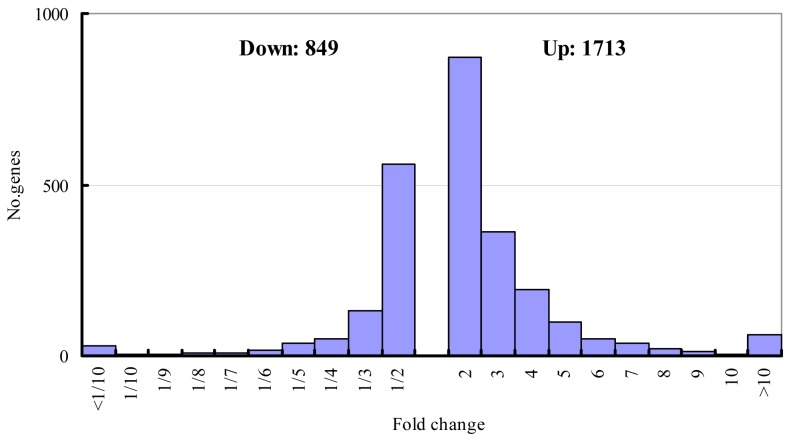
Distribution of genes according to the expression change in response to salt treatment.

According to three screening criteria, a total of 2562 cDNAs were isolated, whose expression changed more than two-fold under stress (Figure 1). Their corresponding genes were defined as salt-responsive. This suggested that approximately 17% of the tested cDNAs were salt-responsive, similar to results reported in wheat [13]. A total of 1713 cDNAs were up-regulated and 849 cDNAs were down-regulated, among which the expression of 1431 cDNAs changed two- to three-fold, 493 cDNAs changed three- to four-fold, 245 cDNAs changed four- to five-fold, and 393 cDNAs changed greater than five-fold (Figure 1). There are accumulated reports that genes which observe a fold-change of two may show no, or very little, differential expression due to the technical problems. The genes which showed changes in expression of over four-fold might be more possible to be involved in salt stress response in *Malus zumi*. As Zhao *et al* [14] describe, The 235 cDNAs which changed four-to five-fold, we functionally classified, according to the Gene Ontology (GO) prediction, into 11 catagories (Table 1): signal transduction (6%), ROS elimination (6%), osmoprotection (5%), cell maintenance and development (6%), photosynthesis (16%), transporter (5%), metabolism (17%), stress tolerance (7%), protein related (7%), others (4%), and unknown (21%) (Table 1). A complete list of the functional classification was shown in Supplementary file (Table S1).

**Figure 2:**
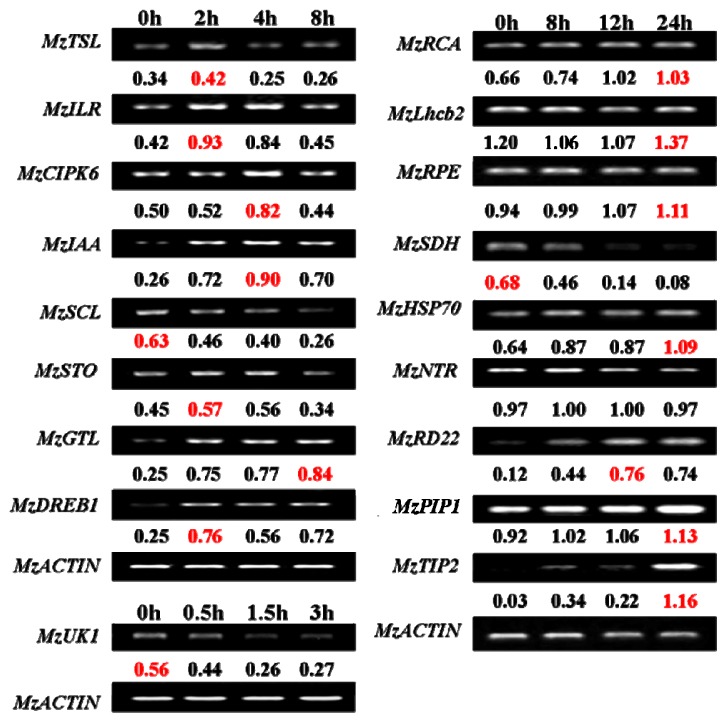
The validation of the expression of 18 selected salt-responsive genes under salt stress by semi-quantitative RT-PCR. The expression levels of tested genes were normalized according to the corresponding actin amplifications, and were presented under the bands. The peak of expression level was colored with red. Except for *MzNTR*, *MzDREB*, *MzCIPK*, and *MzGTL*, the RT-PCR results of 14 tested genes (nearly 78%) had a good correlation with the microarray results.

The microarray results were validated by semi-quantitative RT-PCRs performed on 18 selected genes (Figure 2). *MzTSL*, *MzILR*, *MzCIPK6*, *MzIAA*, *MzSCL*, *MzSTO*, *MzGTL*, and *MzDREB1* were regulatory genes that function in the upstream. *MzRCA*, *MzLhcb2*, *MzRPE*, *MzSDH*, *MzHSP70*, *MzNTR*, *MzRD22*, *MzPIP2*, and *MzTIP2* were functional genes at the downstream level. As the maintaining photosynthesis efficiency under salt stress might be the one of the major mechanisms in *Malus* zumi, we chose three genes (*MzRCA*, *MzLhcb2*, *MzRPE*) involved in photosynthesis. In addition, we also select an unknown gene *MzUK1* for expression confirmation. In the microarray, *MzTSL*, *MzILR*, *MzIAA*, *MzSTO*, *MzRCA*, *MzLhcb2*, *MzHSP70*, *MzNTR*, *MzRD22*, *MzPIP2*, *MzTIP2*, and *MzUK1* were induced by salt stress, when *MzDREB1*, *MzGTL*, *MzSCL*, *MzCIPK6*, and *MzSDH* were suppressed by salt stress (Supplementary file (Table S1)). With the exception of *MzNTR* (sodium dependent phosphate transporter; BankIt1498107), *MzDREB1* (AP2 transcription factor; BankIt1494547), *MzCIPK6* (CIPK6; BankIt1494666), and *MzGTL* (GT-like trihelix DNA-binding protein; BankIt1498102), the RT-PCR results of the other 14 tested genes (78%) were consistent with the microarray data. Similar levels of validation are reported in other microarray studies [15]. This demonstrated the satisfactory quality of our experimental procedures.

**Figure 3:**
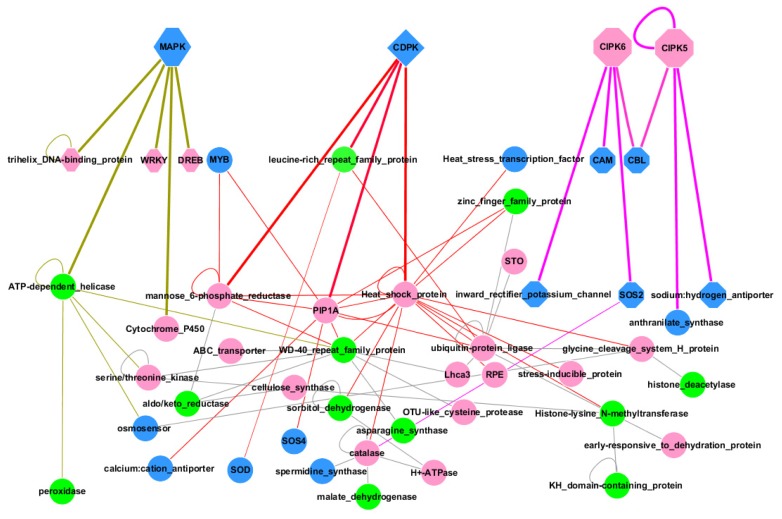
Interaction network of salt-responsive genes in *Malus zumi*. The interactions of salt responsive genes were predicted by their homologs in Arabidopsis. A total of 22 up-regulated genes (**red**), 12 down-regulated genes (**green**) in microarray, and 15 potentially interactive proteins (**blue**), were presented in the interaction network. CDPK appeared as quadrilateral, lines between CDPK and its interactive proteins were magenta; MAPK appeared in hexagon, lines between MAPK and its interactive proteins were khaki; CIPKs appeared in octagon, lines between CIPKs and its interactive proteins were red. The thick lines represented the direct interaction with kinases, the thin lines represented the interaction between proteins. Details of gene information were listed in Supplementary file (Table S2).

Among the eight genes encoding regulatory proteins, *MzSCL* (GRAS family transcription factor; BankIt1494667) was down-regulated and the other seven genes were up-regulated by salt-stress. *MzSCL* belongs to GRAS transcription factor family. Its expression is reduced steadily under salt stress (Figure 2). The seven salt-inducible genes could be divided into two groups according to the time of peak expression. The first group included *MzSTO* (SALT TOLERANCE Homolog Protein; BankIt1493633), *MzDREB1*, *MzILR* (IAA-LEUCINE RESISTANT3; BankIt1495536), and *MzTSL* (Tousled-like Serine/threonine Kinase; BankIt1494726), which responded to salt stress immediately after treatment and reached the peak expression as early as two hours (Figure 2). *MzSTO*, *MzIAA* (BankIt1493693), and *MzGTL* (BankIt1498102) of the second group were up-regulated after treatment and reached the expression peak at four hours (Figure 3). The early response of the tested transcription factors and kinases suggested their possible roles in salt-stress signaling.

We also selected ten genes not involved in transcriptional regulation for RT-PCR analysis. A gene of unknown function (BankIt1498114) and *MzSDH* (sorbitol dehydrogenase; BankIt1495577) were salt suppressed (Figure 2). The gene of unknown function went down sharply and reached the lowest expression at 1.5 h after treatment. In contrast to the previous reports, three genes (*MzRCA*; Ribulose-1, 5-bisphosphate carboxylase; BankIt1495565), *MzLhcb2* (Light-harvesting complex II protein; BankIt1495570), and *MzRPE* (Ribulose-phosphate 3-epimerase; BankIt1498105)) involved in photosynthesis increased their expression steadily after salt treatment. The expression of *MzPIP1* (Plasma Membrane Intrinsic Protein 1; BankIt1494672), *MzTIP2* (Tonoplast Intrinsic Protein 2; BankIt1494673), *MzHSP* (Heat Shock Protein 70; BankIt1498107), and *MzRD22* (RD22; BankIt1495591) were also induced by salt stress. The expression peak of *MzTIP2* appeared at eight hours, while the peak time of the other three genes appeared later than eight hours. A slight increase of expression was found for *MzNTR* under stress.

### 2.2. Promoter Analysis

The promoters of approximately 70 genes were unavailable (or having a promoter region <500 bp), as there are still gaps in apple genome as a result of many areas of repetitive DNA and high heterogeneity [11]. Of the 111 genes with promoters of over 2000 bp, salt-responsive elements were found that included ABRE (abscisic acid responsive element), ARE (anaerobic responsive element), TC-rich repeats (defense and stress responsive element), HSE (heat stress responsive element), DRE (drought responsive element), LTR (low-temperature responsive element), ERE (ethylene responsive element), and MBS (MYB bingding site). The average number of the *cis*-elements per gene changed from 2.43 (ABRE) to 1.0 (DRE). The top three *cis*-elements with high frequency were ABRE, ARE, and MBS (Table 2).

### 2.3. Regulatory Network during Salt Stress

In order to understand the molecular mechanism of apple salt response and explore the protein interaction network of *Malus zumi* under salt stress, an interaction network of salt responsive genes was constructed and the molecular regulatory pathways of apple were alluded to. A total of 155 unigenes, with annotated homologous genes in Arabidopsis, were included in an interaction map using Arabidopsis Interactions Viewer (http://bar.utoronto.ca (accessed on 22 June 2011)) and visualized by Cytoscape 2.6.3 (http://www.cytoscape.org (accessed on 22 June 2011)). Some potential interactive proteins suggested by our salt stress responsive gene set were also included on the map. This suggested that the 58 genes presented in core set of interaction map played critical roles in salt stress response in *Malus zumi*, among which 28 genes were up-regulated and 13 genes were down-regulated from our data (Supplementary file (Table S2)). The interaction of the 58 proteins encoded by these genes (20 regulatory genes and 38 functional genes) will be affected by many aspects of plant activity that contribute together in salt resistance in *Malus zumi* (Figure 4). All of the three primary signaling pathways implicated in salt-stress response (CIPK/SOS, CDPK, and MAPK pathways) appear in apple, but it seems the CDPK pathway plays a central role in this response (Figure 3).

## 3. Discussion

### 3.1. Characteristics of Constructed cDNA Library

A cDNA library was constructed from *Malus zumi* under salt stress by SMART ^™^ cDNA library construction kit. The titer of unamplified cDNA library was 2 × 10^6^ pfu/mL. The recombination efficiency was approximately 90.5%. Colony PCR of 200 randomly selected clones showed that the insertions ranged from 250 bp to 2000 bp, and 115 insertions of different sizes were detected by polyacrylamide gel electrophoresis, which suggested the percentage of uniESTs of our cDNA library was around 58%. The percentage of the insertion sizes between 250 bp to 500 bp was approximately 3%; 500 bp–750 bp, 28%; 750 bp–1000 bp, 36%, and 1.0–2.0 kb, 32%. To assess the percentage of the full-length cDNA insertions in our cDNA library, 30 recombinant clones were sequenced. A total of 16 clones from the tested 30 clones (53%) had full-length cDNA sequences. The 5′UTR (untranslated regions) of full-length clones (0.7 kb–1.6 kb) ranged from 70 bp to 300 bp and 3′UTR ranged from 150 bp to 300 bp. Therefore, the summary statistics of the cDNA library suggested a high quality of cDNA.

### 3.2. Regulatory Network during Salt Stress

An interaction network of salt responsive genes was constructed to describe the molecular mechanism of salt response in *Malus zumi*. Three primary signaling pathways in salt-stress response (CIPK/SOS, CDPK, and MAPK pathways) have been reported in plants [16]. In our network analysis, CDPK proteins appear to play central role in salt response in *Malus zumi*. They were tightly linked to proteins involved in osmolyte metabolism (sorbitol-dehydrogenase acting as key enzyme), and ROS scavenging [17], which are key strategies to defend against salt stress. *CDPK* was not found in our data, but *HSP* (Heat Shock Protein) was linked to *CDPK* and *CIPK*, central in the network, suggesting the possible role of *CIPK* in salt stress of *Malus zumi*. Similarly, *MAPK* were not identified in our study, but *WRKY*, *Trihelix DNA-binding protein* and *AP2* appeared, which are potentially regulated by *MAPKs*. In summary, it seemed CDPK pathway functioned as the primary signaling pathway, *CIPK* and *MAPK* pathway might also be employed during salt stress response in *Malus zumi*.

Although the sensor of salt stress in *Malus zumi* remains unknown, an osmosensor was deduced on the interaction map. This osmosensor was a histidine kinase and is highly homologous to a cytokinin receptor responsive to water deprivation in Arabidopsis [18]. As it is an aspect of salt stress, osmotic stress may be perceived by the osmosensor in *Malus zumi* to trigger the downstream molecular responses. Further efforts should be directed towards discovering the salt-stress sensors and identifying additional signaling components that mediate the salt-stress regulation of the expression and activities of ion transporters.

### 3.3. Salt Stress Signaling

Salt stress signaling played a central role in stress response. Kinases and phosphatases can mediate salt stress response through reversible phosphorylation of transcription factors and other functional genes [4]. A total of 16 genes involved in salt stress signaling were identified, seven genes were induced and nine genes were suppressed under salt stress (Table 3). There were 10 genes encoding transcription factors (zinc finger protein, IAA-LEUCINE RESISTANT3, WRKY, IAA26, Auxin response factor, GRAS family transcription factor, SALT TOLERANCE homolog protein, GT-like trihelix DNA-binding protein and two AP2 transcription factors) and 6 genes encoding kinases (two leucine-rich repeat protein kinase, tousled-like serine/threonine kinase, two CBL-interacting protein kinases and protein kinase family protein).

The WRKY, IAA-LEUCINE RESISTANT3, IAA26, and SALT TOLERANCE homolog protein genes were up-regulated by salt stress while five transcription factors were down-regulated under salt stress in the microarray data. This is the first report of WRKY responding to salt stress in apple. In contrast to a study in Arabidopsis [19], in which the non-induced AtSTO could enhance the salt tolerance of the transgenic plant by its over-expression, the expression of MzSTO increased under salt stress in our microarray result. This is also the first report of salt—inducible auxin responsive protein MzIAA and salt-repressed ARF (Auxin Response Factor) MzARF in apple. In Arabidipsis, IAA26 belongs to Aux/IAA family, which binds ARF to inhibit its activation of downstream auxin response genes for plant growth [20,21]. Our results suggest that they may act collectively to retard growth under salt stress in *Malus zumi*.

In the down-regulated transcription factors, the RT-PCR of *MzSCL* showed good correlation with the microarray result. The two AP2 transcription factors in our research belonged to DREB2 subfamily, which were expected to be salt stress inducible but was repressed in our microarray data. Our RT-PCR showed that they actually enhanced their expression under salt stress.

### 3.4. Analysis of *cis*-Elements

In the promoters of 111 salt responsive genes, ABRE motifs were present most frequently suggesting the major signaling pathways of salt response are probably ABA-dependent [22,23]. HSE were also enriched. The heat-shock protein appeared central of the interaction network, which interacted with HSF (binding to HSE) to release plants from abiotic stress [24,25]. HSP has an important role in salt response as well. MBS is a binding site for MYB transcription factors, which control many abiotic stress responses [26,27]. In addition, ARE motifs are involved in response to anaerobic conditions [28], LTR to low temperature and ERE to ethylene were present indicating the possible roles in low temperature, oxygen shortage, and ethylene response.

### 3.5. Cellular Response Strategies to Salt Stress in *Malus zumi*

The transcriptional changes of salt-responsive genes allude to the signaling and cellular responsive strategies of salt stress in *Malus zumi*. A total of 235 salt-responsive unigenes were obtained whose expression changed more than four-fold after salt treatment. It has been reported in Arabidopsis, rice and other species that osmoprotection, ROS scavenging and ion homeostasis maintenance are the major cellular response strategies for plants under high-salinity environment [2,29,30]. Similar mechanisms were found in apple. Interestingly, possible new mechanism in *Malus zumi* was discovered, that involves sorbitiol. In the response of salt stress, osmoprotection plays a crucial role to maintain the cellular water potential and avoid water loss. A total of four genes with putative functions in osmoprotection were identified in *Malus zumi*. Sorbitol was reported to be an important osmolyte in Rosaceae plants [31]. Salt-inducible *MzS6PDH* (Sorbitol-6-Phosphate Dehydrogenase) and salt- repressed *MzSDH* were identified in our research. *S6PDH* is a well-known rate-limiting enzyme in sorbitol synthesis in mature leaves, while *SDH* converts sorbitol to fructose in sink organs [32]. It was conceivable that the down-regulation of *MzSDH* results in lower sorbitol consumption and up-regulation of *MzS6PDH* leading to more sorbitol synthesis and sorbitol accumulation under salt stress. Three sorbitol transporters of apple, which named *MdSOT3*, *MdSOT4*, and *MdSOT5*, were also induced by drought stress [33]. Mannitol was also a well-known osmolyte in many species [34,35]. Two genes encoding mannose 6-phosphate reductase, the key enzyme in mannitol synthesis, were salt-inducible in our research. The accumulation of sorbitol and mannitol may be critical in osmoprotection under salt stress in *Malus zumi*.

Under a high-salinity environment, water and iron transportation becomes important to controlling ion toxicity and maintaining water relations. An up-regulated *MzNTR* gene encoding ion channel involved in Na^+^/P symport, was identified. This transporter has been documented to enhance salinity tolerance in Arabidopsis [36]. A total of six aquaporins, transmembrane proteins facilitating water transportation across membranes, four plasma membrane intrinsic proteins and two tonoplast intrinsic proteins were obtained, among which five aquaporins were up-regulated by salt stress. *MzPIP1;1* was found to participate in salt stress response in Malus zumi [37].

Several ROS scavengers (glutathione transferase, peroxidase, catalase, and metallothionein) increased their expression. Interestingly an ABC transporter *MzABC*, potentially involved in phenolic transportation into the vacuole was salt responsive. Compared with model plant Arabidopsis, apple had large quantity of phenolic compounds. Some secondary metabolites including phenolic compounds were documented to be involved in ROS scavenging [38,39]. In addition, six proteins identified in our research were reported to be involved in ubiquitin-proteasome pathway in Arabidopsis [40–43], suggesting that ubiquitin-proteasome pathway for protein degradation is salt responsive.

### 3.6. Stable Photosynthesis Suggests a Novel Salt Tolerance Mechanism in *Malus zumi*

In other plant species high salinity inhibits photosynthesis [44–46]. However, in *Malus zumi* a total of 35 genes from the identified 37 unigenes involved in photosynthesis increased their expression under salt stress (Table 4). All the genes encoding light harvesting complex proteins and carbonic anhydrase were induced in microarray result. Salt stress also enhanced the expression of *MzRCA*, which encoded a key enzyme in the Calvin cycle, suggesting this CO_2_-assimilating pathway was stimulated at high salinity. In order to confirm the microarray results, genes functioning in light reaction (*MzLhcb2*), and dark reaction (*MzRPE* and *MzRCA*) were selected for semi-quantitative RT-PCR analysis, which showed consistent results with microarray data (Figure 3). Physiological research has shown stable photosynthesis in *Malus zumi* under salt stress [47]. Conceivably, the up-regulation of genes that play roles in light harvesting and CO_2_ uptake could result in stable photosynthesis under salt stress. Similar physiological responses of photosynthesis to high salinity have been documented in halotolerant plants, such as *Styphnolobium japonicum* and *Atriplex centralasiatica* [48,49]. The ability of *Malus zumi* to maintain normal photosynthetic activity suggests a possible novel salt tolerant mechanism.

### 3.7. Epigenetic and Novel Regulation in Salt Stress Response

Histone modifications play a significant role in stress response [50]. The genes encoding histone deacetylase and histone-lysine *N*-methyltransferase were strongly repressed in our microarray results, suggesting involvement in salt response. The putative interaction between the two proteins and other salt-responsive proteins were presented in the protein interaction network. About 21% (50 unigenes) of salt responsive genes identified in our study were functionally unknown. A total of 24 novel sequences, showing no similarity to any sequence in public databases, were identified. The expression patterns of one functionally unknown gene (*MzUK1*) were confirmed with RT-PCR. These genes, with no known matches in other species or homologous to other genes with known function, may be useful for gene mining to detect unique salt responsive pathways of other woody species.

A total of 16 unigenes involved in other biotic and abiotic stresses were presented in our results, among which five genes were homologous to early drought induced protein, disease resistance protein, pathogenesis-related thaumatin family protein, heat shock protein 70 and UV-induced protein [51–53] (Supplementary file (Table S1)). This suggests possible cross-talk between salt stress and other stresses.

## 4. Experimental Section

### 4.1. Plant Materials

All the seedlings of *Malus zumi* were micropropagated *in vitro* by culturing in MS medium supplemented with 0.5 mg/L BA. Seedlings were transferred to rooting medium (1/2 MS + 1.0 mg/L IAA) when they were at least 3 cm tall. Rooted plants were transplanted into in 1/2 nutrient solution. Nearly one month later, new roots grew out and the rooted plantlets were transferred to complete nutrient solution in a growth chamber (800 μmol·m^−2^·s^−1^ light intensity with 12 h photoperiod) as described by Han *et al.* (1994). The day/night temperature and humidity were maintained at 22~25 °C/ 15~18 °C and 45~50%/60~70%, respectively. Seedlings higher than 10 cm were transferred to nutrient solution supplemented with 150 mM NaCl for salt treatment, nutrient solution without NaCl was used for control. Two biological replicate samples of the leaves and roots were collected at 0 h, 0.5 h, 1 h, 1.5 h, 3 h, 6 h, 12 h, 1 day, 3 day, 5 day, and 7 day after salt treatment, and each sample contained five plants. To prepare RT-PCR materials, Seedlings higher than 10 cm were transferred to nutrient solution supplemented with 150 mM NaCl for salt treatment. Leaves and roots were collected at 0 h, 2 h, 4 h, and 8 h. These materials were stored at −80 °C until use.

### 4.2. RNA Extraction and Construction of the Normalized Full-Length cDNA Library of *Malus zumi*

Total RNA was extracted separately from stored samples, using the CTAB method [54]. Equal amount of total RNAs from salt-treated leaves and roots (0 h, 0.5 h, 1 h, 1.5 h, 3 h, 6 h, 12 h, 1 day, 3 day, 5 day, and 7 day) were mixed to isolate mRNA by PolyAttract mRNA isolation system III (Promega) for cDNA library construction. The leaves and roots from control samples were collected and mixed at the same time point as treated samples. cDNA was purified and normalized to reduce the redundancy. The cDNA library was constructed by using CLONTECH SMART ^™^ cDNA Library Construction Kit. The primary cDNA library was titered and amplified following the protocols. To check the recombination efficiency and insertion size, 200 colony PCRs were conducted to amplify the inserts using the commercial primers (M13-48).

### 4.3. Preparation of Slides for cDNA Microarrays

A total of 15,000 cDNA clones were randomly selected in the cDNA microarray analysis. Insertions of the cDNA clones were amplified by PCR using commercial primers (M13-48). The yield and amplification quality of PCR products were checked on 1% (*w*/*v*) agarose gel. Purified PCR fragments were arrayed from 384-well microtiter plates onto a poly-l-Lys-coated micro slide glass by Cartesian7500 spotting robotics (Cartesian Inc., Newton, MA, USA). Each fragment was dotted twice on the same filter. After spotting, the slides were hydrated for two hours and dried for 0.5 h, which was followed by UV cross-linking and treatment with 0.2% SDS, H_2_O and 0.2% NaBH_4_. The slides were dried again before use.

Salt-responsive cDNA candidates were screened based on these criteria: (1) signal intensity changed more than two-fold on average in both dye-swap experiments; (2) *p*-values of cDNA candidates were less that 0.05; and (3) the spot did not give irregular signals such as due to deformation or dust.

### 4.4. Probe Labeling, Slide Hybridization and Scanning

Two biological replicate samples of the leaves and roots were collected at 0h, 0.5 h, 1 h, 1.5 h, 3 h, 6 h, 12 h, 1 day, 3 day, 5 day, and 7 day after salt treatment. Each sample represented five plants for RNA extraction. The control samples were always same total RNA mixture from five untreated plants. Equal amount of Total RNA extracted from salt-stressed samples were mixed together and labeled with Cy5 by reverse transcription. The cDNA derived from untreated control sample, which was labeled with Cy3 by reverse transcription, was used as the expression reference. The reverse transcription cocktail included 200 U Superscript RNase H^−^ reverse transcriptase (Gibco BRL, Rockville, MD, USA). The Cy3- or Cy5-labeled cDNAs were dissolved in 20 μL solution, containing 5× SSx and 0.2% SDS. After denaturation at 95 °C, chips were placed in ethanol for 30 s. The probes were placed on ice respectively. Hybridizations were performed for 16–18 h at 42 °C in Hybricasette (Shellab Ltd., Cornelius, OR, USA). The slides were then transferred into 0.1% SSC and shaken gently for 20 min. After the slides were rinsed twice, they were spun in a centrifuge and dried by blowing. Dye swap were used in all experiments to compare gene expression changes. The fluorescent signatures were captured using a ScanArray 3000 (GSI; Luminomics, Billercia, MA, USA).

### 4.5. Data Analysis of Microarray Results

Image analysis and signal quantification were performed using ImaGene III software (BioDiscovery, Los Angeles, CA, USA). Background subtraction was carried out using the average of the lowest 5% of spot fluorescence intensities. The log_2_ ratios (signal intensity of Cy5/Cy3) were normalized using the intensity-based Loess method with R language. Wilcoxon Signed Rank Test was applied to compute *p* value using multtest packages in bioconductor (http://www.bioconductor.org/packages/2.12/bioc/html/multtest.html) [55–57]. The False Discovery Rate (FDR) was used for multiple comparison corrections according to Benjamini-Hochberg method [58]. The differentially expressed genes were defined by a log-scale ratio with a corrected *p* value < 0.05. The ratio of intensities between treatment and control indicated gene transcription. When the change of signal intensity between treatment and control exceeded four-fold, the corresponding genes were defined as salt-responsive genes. Then the salt-responsive genes were sequenced (BGI inc. Beishan Street Beishan industrial estate, Yan Tian district, Shenzhen, China) and their homologies were screened with in the GenBank database using BLASTx search program (http://blast.ncbi.nlm.nih.gov/Blast.cgi (accessed on 5 April 2010)). The unigenes were grouped into functional categories according to Gene Ontology (GO) prediction.

### 4.6. RT-PCR Analysis

Reverse transcription was carried out with total RNA (1 μg) isolated from stressed sample/control using SMART^™^ PCR cDNA Synthesis kit following manufacturer’s instructions. The primers for each selected gene were shown in Supplementary file (Table S3). PCRs were performed for one cycle at 94 °C for 3 min, followed by 28 to 38 cycles of 94 °C for 30 s, 52 °C to 53 °C for 30 s, and 72 °C for 40 s, with a final extension of 10 min at 72 °C. *MzACTIN* (AB638619.1) was used as the internal control. The relative amounts of PCR products were quantified by direct scanning of ethidium bromide-stained 1% TAE-agarose gels with Alpha Imager imaging system equipped with the AlphaEaseFC Windows Software. The expression levels of tested genes were normalized according to the corresponding *MzACTIN* amplifications.

### 4.7. Promoter Analysis

A total of 185 arabidopsis genes homologous to annotated apple genes were examined using the GDR database (http://www.rosaceae.org/gb/gbrowse/malus_x_domestica/ (accessed on 10 April 2010)), allowing the promoter sequence of 111genes to be obtained. The 2000bp fragment upstream of the start codon was assumed to be promoter region. The *cis-*elements of promoter sequences were analyzed by PlantCARE (http://bioinformatics.psb.ugent.be/webtools/plantcare/html/ (accessed on 12 April 2010)).

### 4.8. Exploring the Interaction Network

Unigenes identified as responding to salt stress in the microarray experiment were searched against the TAIR database for homologous genes in Arabidopsis. Interactions of these genes were predicted by Arabidopsis Interactions Viewer (http://bar.utoronto.ca) and visualized by Cytoscape 2.6.3 (http://www.cytoscape.org (accessed on 22 June 2011)). The predicted interacting proteins that might play roles in plant salt stress response were then presented in the network.

## 5. Conclusions

We report a global expression profile of salt-responsive genes by microarray analysis in apple rootstock *Malus zumi*. In contrast to previous reports in other species, we found increased expression of genes involved in photosynthesis under salt stress and new mechanisms for ROS scavenging and osmoprotection. An interaction framework of salt stress responsive genes was generated to summarize the salt stress response of *Malus*. Our work contributes to furthering the understanding of salt response mechanisms in apple trees and engineering apple plants with enhanced salt tolerance.

## Supplementary Information



## Figures and Tables

**Table 1:** The functional categorization of the putative salt-responsive genes.

Functional category	Percentage of unigenes (%)
Photosynthesis	16
Transporter	5
Metabolism	17
Stress tolerance	7
Signal transduction	6
Protein related	7
ROS elimination	6
Osmoprotection	5
Cell maintenance and development	6
Others	4
Unknown protein	21

**Table 2:** The presentation of the *cis*-elements in the salt-responsive gene in *Malus zumi*.

*Cis*-element		ABRE	ARE	TC-rich repeats	HSE	DRE	LTR	ERE	MBS
Genes with the *cis*-element among the Regulatory genes		9/14	13/14	10/14	9/14	0/14	8/14	7/14	10/14

Genes with the *cis*-element among the functional genes	ROS elimination	7/10	5/10	8/10	8/10	0/10	4/10	3/10	7/10
	Osmoregulation	7/9	7/9	7/9	2/9	1/9	1/9	2/9	7/9
	Stress tolerance	9/14	10/14	10/14	6/14	2/14	2/14	2/14	12/14
	Photosynthesis	21/26	20/26	17/26	13/26	1/26	12/26	7/26	18/26
	Transporter	7/9	7/9	6/9	7/9	0/9	3/9	1/9	7/9
	Metabolism	17/23	19/23	15/23	16/23	0/23	10/23	8/23	18/23
	Protein related	4/11	10/11	8/11	6/11	0/11	10/11	8/11	18/11
	Cell maintenance and development	2/9	7/9	4/9	7/9	0/9	3/9	1/9	2/9

Average number of the *cis*-elements per gene		2.43	2.24	1.75	2.04	1.00	1.45	1.19	2.10

**Table 3:** Signal elements involved in salt stress response.

Number	Expression a	Putative annotation	Genebank accession	Identities	*p*-value b
Kinase					
1	I	leucine-rich repeat transmembrane protein kinase	NP_199948	64%	5.41 × 10^−5^
1	S	leucine-rich repeat family protein kinase	NP_179336	42%	1.02 × 10^−4^
1	I	tousled-like serine/threonine kinase	NP_568405	82%	3.20 × 10^−5^
1	I	CIPK5	NP_568241	79%	7.74 × 10^−5^
1	S	CIPK6	NP_194825	80%	1.98 × 10^−7^
1	S	protein kinase family protein	NP_194952	50%	2.25 × 10^−5^
Transcription factor					
1	I	IAA-LEUCINE RESISTANT3	NP_200279	89%	4.97 × 10^−4^
1	I	IAA26	NP_188271	80%	3.58 × 10^−6^
1	S	GT-like trihelix DNA-binding protein	NP_177814	37%	3.04 × 10^−6^
1	S	zinc finger (CCCH-type) family protein	NP_200670	59%	8.13 × 10^−5^
1	I	WRKY family transcription factor	NP_001078015	50%	4.52 × 10^−5^
1	S	GRAS family transcription factor	XP_002322514	52%	3.58 × 10^−4^
2	S	AP2 transcription factor	NP_173355	70%	4.26 × 10^−6^
1	I	SALT TOLERANCE homolog protein	NP_849598	68%	5.37 × 10^−7^
1	S	Auxin response factor	NP_182176	70%	2.78 × 10^−3^

a S means suppression, I means induction;

b *p*-value indicates probability of a gene showing significantly differentially expression between salt-treated samples and untreated samples at significance level of 0.05 using FDR correction.

**Table 4:** Genes involved in photosynthesis in salt-stressed *Malus zumi*.

Number	Expression a	Putative annotation	Genebank accession	Identities	*p*-value b
1	S	1-deoxy-D-xylulose-5-phosphate synthase	NP_193291.	41%	5.20 × 10^−4^
1	I	lil3 protein	NP_199522	51%	2.56 × 10^−7^
2	I	photosystem II subunit R	NP_178025	75%	3.43 × 10^−6^
1	I	CHLOROPHYLL A/B BINDING PROTEIN 1	NP_174286	67%	4.38 × 10^−3^
2	I	PSAF (photosystem I subunit F)	NP_174418	72%	3.75 × 10^−5^
1	I	photosystem II CP43 chlorophyll apoprotein	YP_002149729	90%	6.54 × 10^−8^
1	I	photosystem I subunit D-2	NP_171812	72%	8.79 × 10^−4^
1	I	Thylakoid membrane phosphoprotein of 14 kda	NP_566086	64%	9.54 × 10^−7^
1	I	Photosystem I light harvesting complex gene 3	NP_176347	81%	5.37 × 10^−6^
1	I	cytochrome b6	NP_051088	98%	5.86 × 10^−7^
1	I	ATP synthase gamma chain	NP_567265	77%	7.35 × 10^−4^
1	I	NADH dehydrogenase subunit 7	NP_051115	87%	2.36 × 10^−7^
2	I	photosystem II 44 kDa protein	NP_051055	97%	5.38 × 10^−5^
5	I	light-harvesting complex I protein Lhca3	XP_002321218	81%	6.67 × 10^−6^
1	I	Oxygen-evolving enhancer protein	NP_201458	83%	4.31 × 10^−3^
1	I	PSBP-1 (PHOTOSYSTEM II SUBUNIT P-1)	NP_172153	64%	3.34 × 10^−4^
1	I	FERREDOXIN-NADP(+)-OXIDOREDUCTASE 1	NP_201420	76%	4.53 × 10^−5^
1	I	plastid-lipid-associated protein	NP_192311	44%	5.61 × 10^−4^
65	I	ribulose-1,5-bisphosphate carboxylase	CAA79857	91%	5.64 × 10^−6^
1	I	photosystem I P700 chlorophyll a apoprotein A2	NP_051058	98%	6.35 × 10^−3^
7	I	light-harvesting complex II protein Lhcb2	XP_002321186	92%	6.89 × 10^−7^
1	I	light-harvesting complex I protein Lhca4	XP_002330127	75%	8.32 × 10^−6^
1	I	light-harvesting complex I protein Lhca2	XP_002299309	94%	3.57 × 10^−4^
1	I	PHOTOSYSTEM II SUBUNIT O-2	NP_190651	75%	4.25 × 10^−5^
1	S	PsbP domain-containing protein	NP_565131	65%	6.31 × 10^−7^
5	I	light-harvesting complex II protein Lhcb1	XP_002316737	95%	2.58 × 10^−2^
6	I	light-harvesting complex II protein Lhcb6	XP_002303160	82%	5.34 × 10^−5^
1	I	light-harvesting complex II protein Lhcb5	XP_002329192	81%	6.41 × 10^−7^
2	I	photosystem II 22 kDa protein	NP_001150026	91%	3.59 × 10^−3^
2	I	protochlorophyllide reductase	NP_200230	65%	5.34 × 10^−7^
1	I	Clp protease proteolytic subunit 6	NP_563893	45%	4.37 × 10^−5^
2	I	magnesium chelatase H subunit	ACO57443	100%	1.82 × 10^−8^
1	I	Photosystem I P700 chlorophyll A apoprotein	NP_001044491	60%	2.59 × 10^−4^
1	I	carbonic anhydrase	NP_186799	61%	1.56 × 10^−4^
1	I	starch synthase	NP_174566	36%	8.24 × 10^−5^
1	I	glucose-1-phosphate adenylyltransferase	NP_197423	81%	2.34 × 10^−7^
1	I	ribulose-phosphate 3-epimerase, RPE	NP_200949.	83%	5.20 × 10^−4^

a S means suppression, I means induction;

b *p*-value indicates probability of a gene showing significantly differentially expression between salt-treated samples and untreated samples at significance level of 0.05 using FDR correction.
