# Supplementary material for: A Genome-Wide Expression Profile of Salt-Responsive Genes in the Apple Rootstock Malus zumi
Source: Int J Mol Sci. 2013 Oct 18;14(10):21053–70. doi: 10.3390/ijms141021053 (PMC3821658; doi:10.3390/ijms141021053)
Supplement: Supplementary file 1 [file ijms-14-21053-s001.pdf]

Supplementary Information

Table S1. Categories of transcripts significantly regulated in salt-stressed *Malus zumi*.

Number	Expression [a]	Putative Annotation	Genome locus	Genebank Accession	Identities	*p*-value [b]
Signal transduction						
Kinase						
1	I	leucine-rich repeat transmembrane protein kinase	Chr 15	NP_199948	64%	5.41×10^{-5}
1	S	leucine-rich repeat family protein kinase	Chr 15	NP_179336	42%	1.02×10^{-4}
1	I	tousled-like serine/threonine kinase	Chr 11	NP_568405	82%	3.20×10^{-5}
1	I	CIPK5	Chr 1	NP_568241	79%	7.74×10^{-5}
1	S	CIPK6	Chr 2	NP_194825	80%	1.98×10^{-7}
1	S	protein kinase family protein	Chr 6	NP_194952	50%	2.25×10^{-5}
Transcription factor						
1	I	IAA-LEUCINE RESISTANT3	Chr 3	NP_200279	89%	4.97×10^{-4}
1	I	IAA26	Chr 15	NP_188271	80%	3.58×10^{-6}
1	S	GT-like trihelix DNA-binding protein	Chr 15	NP_177814	37%	3.04×10^{-6}
1	S	zinc finger (CCCH-type) family protein	Chr 11	NP_200670	59%	8.13×10^{-5}
1	I	WRKY family transcription factor	Chr 12	NP_001078015	50%	4.52×10^{-5}
1	S	GRAS family transcription factor	Chr 10	XP_002322514	52%	3.58×10^{-4}
2	S	AP2 transcription factor	Chr 15	NP_173355	70%	4.26×10^{-6}
1	I	SALT TOLERANCE homolog protein	Chr 5	NP_849598	68%	5.37×10^{-7}
1	S	Auxin response factor	Chr 7	NP_182176	70%	2.78×10^{-3}

Table S1. *Cont.*

Number	Expression [a]	Putative Annotation	Genome locus	Genebank Accession	Identities	*p*-value [b]
ROS elimination						
1	S	glutathione transferase	Chr 3	NP_850479	62%	3.17×10^{-8}
1	S	peroxidase	Chr 2	NP_201440	71%	4.26×10^{-3}
1	S	peroxidase	Chr 10	NP_197022	54%	3.81×10^{-7}
1	S	peroxidase	Chr 10	NP_192868	64%	4.23×10^{-6}
1	I	catalase	Chr 6	NP_001031791	92%	2.58×10^{-4}
2	S	Metallothionein-like protein type 2	Chr 1	AAC23697	94%	3.26×10^{-7}
1	S	metallothionein-like protein	Chr 11	BAA96449	89%	4.13×10^{-5}
40	I	METALLOTHIONEIN 3	Chr 11	NP_566509	54%	5.05×10^{-3}
1	S	Quinone oxidoreductase-like protein	Chr 9	NP_173786	73%	4.21×10^{-7}
1	I	NADPH quinone oxidoreductase	Chr 2	NP_189427	84%	3.56×10^{-6}
1	I	Cytochrome P450，CPD	Chr 1	NP_001031838	72%	3.78×10^{-8}
1	S	Agglutinin	Chr 6	AAL05954	41%	2.39×10^{-7}
1	I	cytochrome P450	Chr 6	NP_974574	83%	4.25×10^{-7}
2	S	copper ion binding protein	Chr 15	NP_197523	46%	3.89×10^{-3}
Osmoregulation						
1	S	sorbitol dehydrogenase	Chr 1	NP_200010	84%	6.78×10^{-8}
6	I	mannose 6-phosphate reductase	Chr 10	NP_179722.	71%	7.36×10^{-5}
5	I	mannose 6-phosphate reductase	Chr 10	NP_179721	72%	2.21×10^{-4}
1	I	plasma membrane intrinsic protein	Chr 14	BAD14372	90%	1.86×10^{-8}
1	S	plasma membrane intrinsic protein 2	Chr 1	NP_181254	89%	8.91×10^{-7}
2	I	tonoplast intrinsic protein 2	Chr 12	NP_192056	77%	7.35×10^{-9}
3	I	plasma membrane intrinsic protein 2	Chr 12	NP_191042	81%	3.69×10^{-3}
1	I	sodium dependent phosphate transporter	Chr 3	NP_180526	89%	5.34×10^{-4}
1	I	D-sorbitol-6-phosphate dehydrogenase	Chr 10	BAA01853	98%	4.38×10^{-7}
1	S	plasma membrane intrinsic protein 1	Chr 14	NP_191702	95%	5.47×10^{-3}
1	I	tonoplast intrinsic protein 1	Chr11	NP_001045562	73	4.37×10^{-5}

Table S1. *Cont.*

Number	Expression [a]	Putative Annotation	Genome locus	Genebank Accession	Identities	*p*-value [b]
Stress tolerance						
1	S	Mlp-like protein 28,MLP28	Chr 13	NP_001077806	39%	7.35×10^{-4}
1	S	major latex protein	Chr 13	AAQ07269	62%	8.50×10^{-7}
2	I	dehydrin	Chr 2	ABG56268	68%	3.58×10^{-6}
1	I	early drought induced protein	Chr 12	NP_001107634	83%	8.39×10^{-3}
1	S	aldo/keto reductase family protein	Chr 4	NP_001031505	66%	5.32×10^{-7}
5	I	RD22	Chr 5	NP_197943	48%	$3.56\text{-} \times 10^{-8}$
2	I	abscisic stress ripening-like protein	Chr 9	AAL26889	82%	3.85×10^{-4}
1	S	Low-molecular-weight cysteine-rich protein	Chr 16	NP_178319	65%	4.72×10^{-5}
1	S	major allergen mal d 1	Chr 13	AAD26552	100%	5.36×10^{-6}
1	S	polyphenol oxidase 2 precursor	Chr 5	AAK56323	93%	4.38×10^{-7}
2	I	Aldehyde dehydrogenase	Chr 12	NP_567962	56%	7.32×10^{-5}
1	S	disease resistance protein	Chr 6	XP_002318626	44%	3.54×10^{-7}
1	I	pathogenesis-related thaumatin family protein	Chr 9	NP_973870	55%	4.32×10^{-4}
1	I	heat shock protein 70	Chr 1	NP_187864	97%	2.89×10^{-3}
1	I	major allergen Mal d	Chr 13	AAX18313	77%	7.36×10^{-7}
3	S	UV-induced protein uvi31/ BolA family protein	Chr 14	NP_001151829	74%	6.35×10^{-5}
Photosynthesis						
1	S	1-deoxy-D-xylulose-5-phosphate synthase	Chr 16	NP_193291.	41%	5.20×10^{-4}
1	I	lil3 protein	Chr 15	NP_199522	51%	2.56×10^{-7}
2	I	photosystem II subunit R	Chr 10	NP_178025	75%	3.43×10^{-6}
1	I	Chlorophyll a/b binding protein 1	Chr 17	NP_174286	67%	4.38×10^{-3}
2	I	PSAF (photosystem I subunit F)	Chr 12	NP_174418	72%	3.75×10^{-5}
1	I	photosystem II CP43 chlorophyll apoprotein	Chr 11	YP_002149729	90%	6.54×10^{-8}

Table S1. *Cont.*

Number	Expression [a]	Putative Annotation	Genome locus	Genebank Accession	Identities	*p*-value [b]
1	I	photosystem I subunit D-2	Chr 16	NP_171812	72%	8.79×10^{-4}
1	I	Thylakoid membrane phosphoprotein of 14 kda	Chr 10	NP_566086	64%	9.54×10^{-7}
1	I	Photosystem I light harvesting complex gene 3	Chr 17	NP_176347	81%	5.37×10^{-6}
1	I	cytochrome b6	Chr 10	NP_051088	98%	5.86×10^{-7}
1	I	ATP synthase gamma chain	Chr 10	NP_567265	77%	7.35×10^{-4}
1	I	NADH dehydrogenase subunit 7	Chr 17	NP_051115	87%	2.36×10^{-7}
2	I	photosystem II 44 kDa protein	Chr 8	NP_051055	97%	5.38×10^{-5}
5	I	light-harvesting complex I protein Lhca3	Chr 17	XP_002321218	81%	6.67×10^{-6}
1	I	Oxygen-evolving enhancer protein	Chr 8	NP_201458	83%	4.31×10^{-3}
1	I	PSBP-1 (Photosystem II subunit P-1)	Chr 17	NP_172153	64%	3.34×10^{-4}
1	I	Ferredoxin-NADP(+)-oxidoreductase 1	—	NP_201420	76%	4.53×10^{-5}
1	I	plastid-lipid-associated protein	Chr 17	NP_192311	44%	5.61×10^{-4}
65	I	ribulose-1,5-bisphosphate carboxylase	Chr 3	CAA79857	91%	5.64×10^{-6}
1	I	photosystem I P700 chlorophyll a apoprotein A2	Chr 13	NP_051058	98%	6.35×10^{-3}
7	I	light-harvesting complex II protein Lhcb2	Chr 16	XP_002321186	92%	6.89×10^{-7}
1	I	light-harvesting complex I protein Lhca4	Chr 6	XP_002330127	75%	8.32×10^{-6}
1	I	light-harvesting complex I protein Lhca2	Chr 17	XP_002299309	94%	3.57×10^{-4}
1	I	Photosystem II subunit O-2	Chr 8	NP_190651	75%	4.25×10^{-5}
1	S	PsbP domain-containing protein	—	NP_565131	65%	6.31×10^{-7}
5	I	light-harvesting complex II protein Lhcb1	Chr 5	XP_002316737	95%	2.58×10^{-2}
6	I	light-harvesting complex II protein Lhcb6	Chr 2	XP_002303160	82%	5.34×10^{-5}
1	I	light-harvesting complex II protein Lhcb5	Chr 5	XP_002329192	81%	6.41×10^{-7}
2	I	photosystem II 22 kDa protein	Chr 15	NP_001150026	91%	3.59×10^{-3}
2	I	protochlorophyllide reductase	Chr 11	NP_200230	65%	5.34×10^{-7}
1	I	Clp protease proteolytic subunit 6	Chr 10	NP_563893	45%	4.37×10^{-5}

Table S1. *Cont.*

**Number**	**Expression** [a]	**Putative Annotation**	**Genome locus**	**Genebank Accession**	**Identities**	***p*-value** [b]
2	I	magnesium chelatase H subunit	Chr 15	ACO57443	100%	1.82×10^{-8}
1	I	carbonic anhydrase	Chr 9	NP_186799	61%	2.59×10^{-4}
1	I	starch synthase	Chr 7	NP_174566	36%	1.56×10^{-4}
1	I	glucose-1-phosphate adenylyltransferase	Chr 16	NP_197423	81%	8.24×10^{-5}
1	I	ribulose-phosphate 3-epimerase,RPE	Chr 14	NP_200949.	83%	2.34×10^{-7}
Transporter						
2	S	lipid transfer protein	Chr 4	NP_568699	50%	4.21×10^{-8}
4	S	protease inhibitor/seed storage/lipid transfer protein family protein	Chr 13	NP_190966.	61%	3.25×10^{-5}
1	S	Multidrug resistance-associated protein 3, ABC transporter family protein	Chr 14	NP_187915	80%	5.63×10^{-4}
3	I	White-brown complex homolog protein 23, ABC transporter family protein	Chr 14	NP_001031843	62%	3.84×10^{-7}
1	I	H+/oligopeptide symporter POT family	Chr 17	NP_188804	77%	5.37×10^{-3}
1	I	protease inhibitor/seed storage/lipid transfer protein	Chr 17	NP_566036	76%	2.34×10^{-5}
3	S	Protease inhibitor/seed storage/lipid transfer protein	Chr 13	NP_568160	75%	7.86×10^{-4}
3	I	nonspecific lipid transfer protein 1	Chr 11	NP_181388	45%	8.65×10^{-8}
4	I	proton-dependent oligopeptide transport (POT) family protein	Chr 16	NP_177024	70%	2.41×10^{-3}
1	I	Non-specific lipid-transfer protein 4	Chr 11	NP_568904	46%	4.36×10^{-4}
2	I	hydrogen ion transporting ATP synthase	Chr 17	NP_001044491	60%	6.53×10^{-4}

Table S1. *Cont.*

Number	Expression [a]	Putative Annotation	Genome locus	Genebank Accession	Identities	*p*-value [b]
Metabolism						
1	I	phosphatidylcholine-sterol O-acyltransferase	Chr 2	NP_196868	78%	3.54×10^{-3}
1	I	Flavonol synthase/flavanone 3-hydroxylase	Chr 5	EEF39692	77%	5.21×10^{-7}
1	I	triose-phosphate isomerase	Chr 16	NP_001077931	86%	3.57×10^{-6}
9	I	fructose-bisphosphate aldolase	Chr 13	NP_568049	82%	5.64×10^{-8}
1	I	fructose-bisphosphate aldolase	Chr 15	NP_175032	94%	3.98×10^{-5}
6	I	fructose-bisphosphate aldolase	Chr 13	NP_974710	80%	2.34×10^{-4}
8	I	glyceraldehyde-3-phosphate dehydrogenase	Chr 15	NP_180004	89%	7.31×10^{-3}
1	I	acyl-activating enzyme 11	Chr 9	NP_176786	62%	5.39×10^{-5}
1	I	carotenoid cleavage dioxygenase	Chr 16	ABY47995	74%	6.28×10^{-3}
1	S	Formate dehydrogenase	Chr 17	NP_196982	89%	7.65×10^{-5}
1	S	Beta-amylase	Chr 15	NP_567523	73%	5.43×10^{-7}
1	I	acyl-ACP thioesterase	Chr 5	NP_193041	64%	3.25×10^{-3}
1	I	ATP synthase CF0 subunit IV	Chr 16	YP_001806690	79%	2.31×10^{-5}
1	I	ATP synthase	Chr 1	AAM64665	70%	3.56×10^{-7}
1	I	NADH dehydrogenase I subunit N	Chr 11	ACG27527	69%	6.54×10^{-6}
1	I	dehydrodolichyl diphosphate synthase	Chr 12	NP_001077518	45%	9.21×10^{-3}
1	S	alcohol dehydrogenase	Chr 13	NP_173786	78%	8.37×10^{-7}
1	I	Lipoxygenase	Chr 7	NP_177396	52%	6.53×10^{-5}
1	S	RNA binding protein, TGH	Chr 6	NP_197699	55%	6.28×10^{-7}
1	S	Arginine/serine-rich zinc knuckle-containing protein	Chr 5	NP_850280	75%	2.37×10^{-5}
1	I	XRCC4 homolog	Chr 13	NP_188951	72%	7.35×10^{-7}
1	I	DNA-damage-repair/toleration protein	Chr 8	NP_173459	63%	8.51×10^{-4}
1	S	DNA double-strand break repair rad50 ATPase	—	XP_002525969	49%	5.36×10^{-3}
1	I	eukaryotic translation initiation factor 5A	Chr 2	AAL10404	95%	3.64×10^{-5}

Table S1. *Cont.*

Number	Expression [a]	Putative Annotation	Genome locus	Genebank Accession	Identities	*p*-value [b]
1	I	eukaryotic translation initiation factor 2B family protein	Chr 14	NP_198680	70%	5.38×10^{-8}
1	I	ATP synthase CF0 C subunit	Chr 15	NP_051046	98%	6.94×10^{-4}
1	I	NAD-dependent epimerase/dehydratase	Chr 12	NP_001148959	85%	5.75×10^{-3}
2	I	(S)-2-hydroxy-acid oxidase	Chr 5	NP_850585	93%	5.34×10^{-5}
1	I	(S)-2-hydroxy-acid oxidase	Chr 10	NP_188059	91%	6.28×10^{-4}
1	S	terpene synthase/cyclase family protein	Chr 3	NP_197784	47%	5.64×10^{-7}
1	I	alcohol acyl transferase	Chr 13	AAU14879	88%	6.37×10^{-7}
1	I	3-oxo-5-alpha-steroid 4-dehydrogenase family protein	—	NP_177403	42%	2.89×10^{-4}
1	S	malate oxidoreductase	Chr 7	NP_191966	71%	4.13×10^{-4}
1	S	Nudix hydrolase homolog gene	Chr 6	NP_565273	52%	4.28×10^{-7}
1	S	ATP-dependent helicase	Chr 12	NP_188307	60%	1.89×10^{-3}
1	I	haloacid dehalogenase-like hydrolase family protein	Chr 14	NP_181658	56%	6.14×10^{-4}
1	I	thiamin diphosphokinase	Chr 11	NP_850424	66%	7.54×10^{-4}
1	I	myo-inositol-1-phosphate synthase	Chr 6	NP_195690	60%	8.69×10^{-3}
2	I	inositol-3-phosphate synthase	Chr 6	NP_196579	91%	1.47×10^{-7}
2	S	chalcone synthase	Chr 2	NP_196897	69%	6.28×10^{-8}
Protein related						
1	S	histone deacetylase	Chr 9	XP_002326411	61%	3.49×10^{-3}
1	S	Histone-lysine N-methyltransferase	Chr 15	NP_849969.	68%	5.31×10^{-4}
6	I	OTU-like cysteine protease family protein	Chr 11	NP_175482	75%	6.79×10^{-7}
1	I	glycine cleavage system H protein	Chr 6	NP_174525	85%	8.23×10^{-6}
1	I	Ubiquitin carboxyl-terminal hydrolase 23	Chr 14	NP_568873	50%	5.32×10^{-5}
1	I	Peptidase M3 family protein	Chr 1	NP_569013	58%	1.03×10^{-4}
1	S	asparagine synthase (glutamine-hydrolyzing)	Chr 11	AAO38524	75%	3.05×10^{-3}

Table S1. *Cont.*

Number	Expression a	Putative Annotation	Genome locus	Genebank Accession	Identities	*p*-value [b]
1	S	glutamate-ammonia ligase	Chr 14	NP_001051067	91%	3.60×10^{-4}
2	I	alanine aminotransferase2	Chr 13	NP_177215	92%	9.02×10^{-6}
1	S	alanine aminotransferase1	Chr 9	NP_173173	86%	2.34×10^{-7}
3	I	Alanine-2-oxoglutarate aminotransferase1	Chr 13	NP_001031083	92%	6.12×10^{-5}
1	I	ubiquitin-conjugating enzyme E2	Chr 4	NP_567791	96%	8.05×10^{-4}
1	S	Defective in cullin neddylation protein	Chr 1	EEF35070	77%	7.31×10^{-4}
1	S	ubiquitin interaction motif-containing protein	Chr 10	NP_175034.	74%	6.03×10^{-3}
1	S	Subtilase	Chr 17	NP_564107	49%	3.04×10^{-3}
1	I	SIT4 phosphatase-associated family protein	Chr 5	NP_174335	79%	7.25×10^{-5}
1	I	reticulon family protein	Chr 3	NP_176592	57%	6.00×10^{-3}
Cell maintenance and development						
1	I	60S ribosomal protein L8	Chr 14	NP_195336	91%	5.31×10^{-4}
1	I	40S ribosomal protein S23	Chr 3	NP_001131287.	97%	5.14×10^{-7}
1	I	30S ribosomal protein S11	Chr 10	NP_564385	54%	3.56×10^{-3}
1	S	40s ribosomal protein s13	Chr 2	NP_567151	94%	8.34×10^{-7}
2	I	30S ribosomal protein S16	—	NP_200504	91%	9.01×10^{-6}
1	I	tropomyosin-related	Chr 2	NP_199627	62%	1.27×10^{-3}
2	S	pollen Ole e 1 allergen and extensin family protein	Chr 17	NP_197082	56%	5.39×10^{-4}
1	I	dynein light chain	Chr 3	NP_194466	71%	4.23×10^{-7}
1	S	chromosome-associated kinesin	Chr 8	NP_200901	76%	2.08×10^{-6}
1	I	pollen Ole e 1 allergen and extensin family protein	Chr 9	NP_197082	62%	2.38×10^{-4}
1	S	pectinesterase	Chr 5	NP_172624	37%	5.64×10^{-3}
1	I	cellulose synthase	Chr 8	EEF33090	65%	4.30×10^{-6}
1	S	O-methyltransferase	Chr 5	NP_195242	50%	2.08×10^{-5}
1	S	O-methyltransferase	Chr 16	XP_002302676	57%	2.14×10^{-4}

Table S1. *Cont.*

Number	Expression [a]	Putative Annotation	Genome locus	Genebank Accession	Identities	*p*-value [b]
Others						
1	S	KH domain-containing protein	Chr 3	NP_200425	84%	3.56×10^{-7}
1	S	WD-40 repeat family protein	Chr 10	NP_566644	75%	8.30×10^{-5}
1	S	VQ motif-containing protein	Chr 14	NP_195485	50%	2.37×10^{-6}
1	I	NC domain-containing protein-related	Chr 1	NP_563621	78%	2.68×10^{-3}
1	I	ABI3-interacting protein	Chr 6	BAD19899	61%	8.50×10^{-7}
1	I	GTP binding protein	Chr 11	EEF44801	53%	7.21×10^{-6}
1	S	DNA-binding protein	Chr 4	NP_001151240	81%	3.45×10^{-7}
1	I	Protein pob	Chr 10	EEF41790	90%	5.31×10^{-8}
1	I	peroxisomal membrane protein-related	Chr 3	NP_564616	40%	2.75×10^{-5}
1	I	FG-GAP repeat protein	—	XP_001265249	59%	5.31×10^{-4}
1	I	ankyrin repeat-containing protein	Chr 3	EEF27218	50%	3.60×10^{-3}
Unknown						
1	I	hypothetical protein	Chr 17	YP_173415.	95%	8.14×10^{-4}
1	S	unknown protein	Chr 10	NP_194575	44%	7.02×10^{-6}
1	S	unknown protein	Chr 7	NP_191604	61%	$\times 10^{-7}$
1	S	unknown protein	Chr 2	NP_566044.	38%	3.62×10^{-8}
1	S	unknown protein	Chr 9	NP_180647.	59%	3.74×10^{-5}
1	S	unknown protein	Chr 1	NP_569010.	31%	1.05×10^{-4}
1	S	unknown protein	Chr 2	NP_192113	38%	8.23×10^{-6}
1	I	unnamed protein	—	CAO21969	32%	9.79×10^{-5}
1	S	unknown protein	Chr 12	NP_564513	58%	3.59×10^{-4}
1	S	unknown protein	Chr 7	NP_182138	42%	2.74×10^{-5}
1	S	unknown protein	Chr 5	NP_176367	57%	3.85×10^{-6}
1	I	unknown protein	Chr 9	NP_193006	65%	5.23×10^{-7}
1	I	unknown protein	Chr 15	NP_568382	47%	4.39×10^{-8}
1	I	unnamed protein	Chr 3	CAO21221	74%	3.23×10^{-5}
1	S	Predicted protein	Chr 1	XP_002302313	50%	5.04×10^{-4}

Table S1. *Cont.*

Number	Expression a	Putative Annotation	Genome locus	Genebank Accession	Identities	*p*-value [b]
1	S	unknown protein	Chr 17	NP_172138	57%	4.65×10^{-3}
1	I	hypothetical protein	Chr 7	XP_002282574	70%	6.37×10^{-7}
1	I	predicted protein	Chr 6	XP_002298251	59%	1.85×10^{-8}
1	I	hypothetical protein	Chr 4	XP_002264843	70%	2.13×10^{-6}
1	I	predicted protein	Chr 3	XP_002303990	90%	5.36×10^{-6}
1	S	unnamed protein product	—	BAE89103	100%	5.98×10^{-7}
1	I	predicted protein	Chr 12	XP_002305421	51%	2.30×10^{-4}
1	S	hypothetical protein	Chr 12	XP_002266957	55%	6.67×10^{-7}
1	I	predicted protein	Chr 8	XP_002330640	45%	8.32×10^{-6}
1	I	predicted protein	Chr 9	XP_002305896	60%	3.54×10^{-4}
1	I	hypothetical protein	Chr 11	XP_002273979	53%	2.70×10^{-3}

[a] S means suppression, I means induction; [b] P-value indicates probability of a gene showing significantly differentially expression between salt-treated samples and untreated samples at significance level of 0.05 using FDR correction.

Table S2. Gene homologs of unigenes in Arabidopsis in the interaction network.

Microarray Gene AGI Code [a]	Microarray Gene	Interaction protein AGI Code [a]	Interaction protein Annotation
At1g61520	LHCA3	At2g16740	ubiquitin-protein ligase
At1g61520	LHCA3	At2g22040	WD-40 repeat family protein
At3g13080	ABC transporter family protein	At5g54200	WD-40 repeat family protein
At3g16840	ATP-dependent helicase	At1g30270	serine/threonine kinase
At3g16840	ATP-dependent helicase	At1g59760	ATP-dependent helicase
At3g16840	ATP-dependent helicase	At2g46070	MAPK
At3g16840	ATP-dependent helicase	At5g15550	WD-40 repeat family protein
At3g16840	ATP-dependent helicase	At2g01830	osmosensor
At2g37770	aldo/keto reductase family protein	At2g21250	mannose 6-phosphate reductase
At2g37770	aldo/keto reductase family protein	At3g18860	WD-40 repeat family protein
At2g37770	aldo/keto reductase family protein	At4g32410	cellulose synthase
At2g17440	leucine-rich repeat family protein	At1g55860	ubiquitin-protein ligase
At2g17440	leucine-rich repeat family protein	At2g41860	CDPK
At2g17440	leucine-rich repeat family protein	At3g10920	SOD
At3g12580	Heat shock protein	At1g62740	stress-inducible protein
At3g12580	Heat shock protein	At2g16740	ubiquitin-protein ligase
At3g12580	Heat shock protein	At2g32850	protein kinase family protein
At3g12580	Heat shock protein	At1g54270	ATP-dependent helicase
At3g12580	Heat shock protein	At3g07200	zinc finger family protein
At3g12580	Heat shock protein	At3g12580	Heat shock protein
At3g12580	Heat shock protein	At3g61430	PIP1A
At3g12580	Heat shock protein	At5g03720	Heat shock transcription factor
At3g12580	Heat shock protein	At4g35310	CDPK
At3g12580	Heat shock protein	At5g54200	WD-40 repeat family protein
At1g06040	STO	At2g32950	ubiquitin-protein ligase
At1g50670	OTU-like cysteine protease family protein	At3g18860	WD-40 repeat family protein
At1g32470	glycine cleavage system H protein	At1g27320	osmosensor
At1g32470	glycine cleavage system H protein	At3g18520	histone deacetylase
At1g32470	glycine cleavage system H protein	At5g09590	Heat shock protein
At1g32470	glycine cleavage system H protein	At5g53300	ubiquitin-protein ligase
At3g47340	asparagine synthase	At4g18900	WD-40 repeat family protein
At4g27960	ubiquitin-protein ligase	At1g26830	ubiquitin-protein ligase
At4g27960	ubiquitin-protein ligase	At3g10910	zinc finger family protein
At4g27960	ubiquitin-protein ligase	At4g22120	early-responsive to dehydration protein
At4g35090	catalase	At1g20260	H+-ATPase
At4g35090	catalase	At2g33210	Heat shock protein
At4g35090	catalase	At3g47520	malate dehydrogenase

Table S2. *Cont.*

Microarray Gene AGI Code [a]	Microarray Gene	Interaction protein AGI Code [a]	Interaction protein Annotation
At4g35090	catalase	At4g33950	serine/threonine kinase
At4g35090	catalase	At4g35090	catalase
At4g35090	catalase	At5g53120	spermidine synthase
At5g51970	sorbitol dehydrogenase	At3g28715	H+-ATPase
At5g51970	sorbitol dehydrogenase	At4g37910	heat shock protein
At5g51970	sorbitol dehydrogenase	At5g51970	sorbitol dehydrogenase
At2g21250	mannose 6-phosphate reductase	At1g80030	Heat shock protein
At2g21250	mannose 6-phosphate reductase	At2g21250	mannose 6-phosphate reductase
At2g21250	mannose 6-phosphate reductase	At3g61430	PIP1A
At2g21250	mannose 6-phosphate reductase	At4g04700	CDPK
At2g21250	mannose 6-phosphate reductase	At5g45420	MYB
At2g21250	mannose 6-phosphate reductase	At5g54200	WD-40 repeat family protein
At3g61430	PIP1A	At1g55730	calcium:cation antiporter
At3g61430	PIP1A	At2g16740	ubiquitin-protein ligase
At3g61430	PIP1A	At3g47940	Heat shock protein
At3g61430	PIP1A	At4g35310	CDPK
At3g61430	PIP1A	At5g06420	zinc finger family protein
At3g61430	PIP1A	At5g37850	SOS4
At3g61430	PIP1A	At5g45420	MYB
At3g61430	PIP1A	At5g54200	WD-40 repeat family protein
At5g05690	Cytochrome P450	At4g11330	MAPK
At3g19590	WD-40 repeat family protein	At2g20635	serine/threonine kinase
At5g56140	KH domain-containing protein	At3g04380	histone-lysine N-methyltransferase
At5g56140	KH domain-containing protein	At5g46190	KH domain-containing protein
At2g17900	Histone-lysine *N*-methyltransferase	At5g26751	serine/threonine kinase
At2g17900	Histone-lysine *N*-methyltransferase	At5g46190	KH domain-containing protein
At2g17900	Histone-lysine *N*-methyltransferase	At5g52640	Heat shock protein
At5g20930	serine/threonine kinase	At5g20930	serine/threonine kinase
At5g10930	CIPK5	At1g25155	anthranilate synthase
At5g10930	CIPK5	At2g31910	sodium:hydrogen antiporter
At5g10930	CIPK5	At4g26570	CBL
At5g10930	CIPK5	At5g10930	CIPK5

Table S2. *Cont.*

Microarray Gene AGI Code [a]	Microarray Gene	Interaction protein AGI Code [a]	Interaction protein Annotation
At4g30960	CIPK6	At2g26650	inward rectifier potassium channel
At4g30960	CIPK6	At3g43810	CAM
At4g30960	CIPK6	At5g24270	CBL
At1g76880	trihelix DNA-binding protein	At1g59580	MAPK
At1g76880	trihelix DNA-binding protein	At1g76880	trihelix DNA-binding protein
At2g37260	WRKY	At4g11330	MAPK
At1g19210	DREB	At4g11330	MAPK
At1g06040	STO	At2g32950	ubiquitin-protein ligase

[a] Arabidopsis Genome Initiative.

Table S3. Primers used in semi-quantitative RT-PCR.

Gene	Primer sequence (5'→3')	T_m (°C)	Cycles
MzTSL	F:AAGCAGAGTTACATACGGCA	53	28
	R: TAATGATCCTAGCTTCCCTC		
MzILR	F:CCTGGCTTTTTGCCTCCC	53	28
	R:CGGTGGGCGGAGTACATG		
MzCIPK	F:CGAGCTCCACGAGGTCAT	53	28
	R:AAGTTGCCGTCCTGGTCTA		
MzIAA	F:TTGGGTTCCTTTAGTAGATT	53	28
	R:TAACCTCTTCAAATCTCAAA		
MzSCL	F:CAGATAGATGCCTACGGG	53	28
	R:CACCTTGCCATACCACTC		
MzSTO	F:CAGAGCCCTCATTTGTCA	53	28
	R:ATCGTCCTCCAGCATTTC		
MzGTL	F:ATGTCCTTACTTTCACCTGCTA	53	28
	R:GCCTCACCATCAGAGGTAC		
MzDREB1	F:GCACTTTCACTGCATGTCAA	52	32
	R:TTCCGACACCCATTTGC		
MzRCA	F:TGTAGTCGCAGCTCCTCA	52	32
	R:CATCTGTCCTGGTTGGTTT		
MzLhcb2	F:ATGGCAACCTCTGCTATCC	52	30
	R:GCAGTGTCCCATCCGTAG		
MzRPE	F:TCGGCTGCTTCACTTTGT	52	30
	R:CACAACCTGCCAACTCTACA		
MzSDH	F:AGGGAGGTTGATGTGGTT	52	38
	R:GTAGAAACCAGAACTTTGTCC		
MzHSP70	F:CTGCGATTCTTTGCTTGTT	52	31
	R:CAGTAAACGCCACGTAAGA		
MzNTR	F:CCCCGCTTTCTTCCTAAC	52	34
	R:TACACCTGCCAGCACTCC		

Table S3. *Cont.*

Gene	Primer sequence (5'→3')	Tm (℃)	Cycles
MzRD22	F:ATGCTGCTCTACCACCTCA	52	32
	R:TTGACACCACCCTTTCCTA		
MzPIP1;1	F:GTTACACCAAGGGTCAAGGA	53	32
	R:CTGGGACTGGAAAGGGAT		
MzTIP2	F:CTGCTTTCTCCCTATCGTC	53	32
	R:AGTGGTTGTCCCATGTCC		
MzUK1	F:GGTGAATCCGATTAGTAATGGTG	53	28
	R:CCTAATTCCCAAGTAACCTCTTCT		

© 2013 by the authors; licensee MDPI, Basel, Switzerland. This article is an open access article distributed under the terms and conditions of the Creative Commons Attribution license (http://creativecommons.org/licenses/by/3.0/).
